# Immunoregulation via Cell Density and Quorum Sensing-like Mechanisms: An Underexplored Emerging Field with Potential Translational Implications

**DOI:** 10.3390/cells11152442

**Published:** 2022-08-06

**Authors:** Adrian A. Naoun, Itay Raphael, Thomas G. Forsthuber

**Affiliations:** 1 Department of Molecular Microbiology and Immunology, University of Texas at San Antonio, San Antonio, TX 78249, USA; 2 Department of Neurological Surgery, School of Medicine, University of Pittsburgh, Pittsburgh, PA 15217, USA

**Keywords:** quorum sensing, immune system, cytokines, T cell homeostasis, macrophage

## Abstract

Quorum sensing (QS) was historically described as a mechanism by which bacteria detect and optimize their population density via gene regulation based on dynamic environmental cues. Recently, it was proposed that QS or similar mechanisms may have broader applications across different species and cell types. Indeed, emerging evidence shows that the mammalian immune system can also elicit coordinated responses on a population level to regulate cell density and function, thus suggesting that QS-like mechanisms may also be a beneficial trait of the immune system. In this review, we explore and discuss potential QS-like mechanisms deployed by the immune system to coordinate cellular-level responses, such as T cell responses mediated via the common gamma chain (γc) receptor cytokines and the aryl hydrocarbon receptors (AhRs). We present evidence regarding a novel role of QS as a multifunctional mechanism coordinating CD4^+^ and CD8^+^ T cell behavior during steady state and in response to infection, inflammatory diseases, and cancer. Successful clinical therapies such as adoptive cell transfer for cancer treatment may be re-evaluated to harness the effects of the QS mechanism(s) and enhance treatment responsiveness. Moreover, we discuss how signaling threshold perturbations through QS-like mediators may result in disturbances of the complex crosstalk between immune cell populations, undesired T cell responses, and induction of autoimmune pathology. Finally, we discuss the potential therapeutic role of modulating immune-system-related QS as a promising avenue to treat human diseases.

## 1. Introduction

The lifestyle of unicellular organisms, such as bacteria and fungi, was initially envisioned as autonomous and reclusive, whereas more sophisticated multicellular organisms were understood to operate by collectively sensing and reacting to signals in a tissue-collaborative fashion. However, it was later observed that substantial increases in bacterial population density resulted in the accumulation of soluble extracellular inducers, which, above a minimal threshold, facilitated synchronized gene expression patterns to regulate behaviors such as biofilm formation, virulence factor expression, motility, and bioluminescence [1,2,3]. These phenomena have emerged as a density-dependent microbial communication mechanism mediated by molecules known as autoinducers and have been termed quorum sensing (QS) [4]. Recent evidence showed that autoinducer-mediated communication is also observed in other organisms, such as fungi [5], and suggested that QS regulation or similar mechanisms may also govern the mammalian immune system [6]. While the mammalian immune system was long known to elicit synergistic responses, for example, against invading pathogens, the concept of QS-like behavior may add a new layer to our understanding of the underlying mechanisms.

The immune system has a fundamental role in protecting a host from microbial invasion [7,8]. Two arms of the immune system are engaged at different stages of infections and collaborate to defend against microbial invaders [7,9]. Initial responses are non-specific and facilitated by the innate immune system cells such as neutrophils, macrophages, and dendritic cells. Subsequently, the adaptive immune system is engaged via T and B lymphocytes [9]. 

These collaborative efforts include recruitment of specific immune cell populations to infected or inflamed tissues, recognition, destruction, and antigen presentation of pathogens and coordination of the proper type of immune responses, e.g., antibody class or cytokines for optimized pathogen defense [10]. However, every population of immune cells engaged in this process exhibits some degree of diversity at the level of immune cell subpopulations (e.g., M1 vs. M2 macrophages, different DC populations), or T cell receptors (TCRs) and B cell receptors (BCRs) determined by the clonality of T and B lymphocytes [11,12]. Evidently, innate and adaptive immunity cooperate on multiple levels, for instance, via recruiting adaptive immune cells by cytokines, chemokines, and the complement system provided by the innate immune system [13,14]. However, while factors such as chemokines provide strong, overarching signals recruiting immune cells to infected and/or inflamed tissues, an additional layer of spatiotemporal fine-tuning and collective coordination may be achieved by population-level QS-like cues [6,15]. Along these lines, a central role for QS is based on the capacity of immune cells such as T cells, B cells, and macrophages to sense their own cell density within a certain tissue area, as well as to sense the density of other cell populations in proximity to this region and to coordinate cell density during homeostasis and inflammatory conditions. QS in the immune system may utilize soluble mediators (e.g., cytokines, chemokines, certain metabolites) as indicators of cell density, and these mediators may act on different immune cell types directly, or indirectly via cellular intermediaries. The primary task of QS is thereby assumed to facilitate defense against microbial invasion; however, disruption of immune QS may potentially result in immune-related disorders [15]. 

Mammalian immunoregulation via a QS-like behavior shows parallels to the bacterial counterparts since many transcriptional alterations are also cell-density-dependent. For example, secretion of cytokines and chemokines in sufficient concentrations facilitates collective receptor-mediated responses to modulate host cell behavior, hence mimicking bacterial autoinducer detection and adaptation to dynamic environmental cues. Thus, eliciting coordinated cellular responses is imperative to regulate tissue and organ homeostasis and function, seemingly coordinated across the cellular up to the entire system levels; therefore, we posit that QS-like behavior may play an important role in immune defense and potentially immune-related dysfunction. In the following sections, we discuss evidence of QS-like behavior in the lymphoid and myeloid lineages and the application of such QS-like mechanisms to design new therapies for cancer and inflammatory diseases.

## 2. Quorum Sensing-like Mechanisms of T Cells and Regulation of the Adaptive Immune System

Clonally expanded T and B cell populations can be conventionally subclassified based on shared properties such as surface marker expression and cytokine profiles [16,17,18]. Nevertheless, on a single-cell level, functionality is rendered by a unique TCR or BCR encoding each cell’s specificity and affinity for antigens, including microbial and endogenous antigenic epitopes [19,20,21,22]. After activation, B cells may differentiate into subpopulations such as germinal center B cells, plasmablasts, and plasma cells, whereas effector T cells can assume Th1, Th2, Th17, or other T cell phenotypes. Contrary to expectations, these B or T cell subpopulations seem superficially alike but may be phenotypically and functionally variable. For instance, a T cell may fall into the Th17 category based on its secretion of IL-17, but not all Th17 cells specific for a shared cognate antigen exhibit identical activation profiles. Even clonal T cells with identical TCR may exhibit different activation profiles depending on their activation state [22,23,24].

The parameters defining individual versus communal T or B cell behavior may be inherently attributable to the anatomical microenvironment; however, local lymphocyte collaboration should confer host benefits under physiological and inflammatory conditions. Along these lines, single-cell RNA sequencing (scRNA-seq) analyses evaluating resting and TCR-stimulated human T cells from primary, secondary, or mucosal tissue sites revealed conserved gene expression signatures within anatomical compartments [25]. 

Density of CD4^+^ and CD8^+^ T cells during antigen stimulation may also serve as an additional mechanism regulating the magnitude of T cell activation and/or differentiation in a local milieu. For example, in vivo studies showed that the function and phenotype of CD8^+^ T cells is dictated by the precursor cell pool present during priming [26,27,28]. Moreover, the trajectory of CD4^+^ T cell differentiation is modulated by the number of locally interacting cells rather than the initial cell concentration [29]. Intercellular communication failures may potentially result in immune aberrations, promoting chronic inflammatory or autoimmune conditions.

### 2.1. Common-Gamma Chain (γc) Receptor and Cytokines as QS-like Mechanism for Coordinating T Lymphocyte Responses and Homeostasis

QS in bacteria is defined as an autocrine/paracrine density-dependent mechanism leading to rapid cellular function adaptation [4]. Similarly, lymphocytes have the capability to signal to each other via both autocrine and paracrine mediators, including cytokines, which then results in functional changes and coordinated function [30]. This is important, for example, to maintain the proper homeostasis of naïve and memory T cell populations in an individual [31]. Interestingly, homeostatic regulation of lymphocyte density via proliferation, survival, and metabolic reprograming is a function reminiscent of bacterial QS. 

Conceptually, homeostatic regulatory mechanisms serve to keep the total size of both the naïve and memory T cell pool relatively constant [32,33,34], and one of the critical mechanisms relies on signals provided via members of the common gamma chain (γc) cytokine-receptor family and their ligands [35,36], which are commonly referred to as the γc family of cytokines [37,38,39]. As a key member of this family, IL-2/IL-2R controls the differentiation and homeostasis of both pro- and anti-inflammatory T cells and is central to determining key aspects of immune regulation [40]. IL-7 and IL-7R, two other members of the γc family of cytokines, primarily govern survival, whereas IL-15/IL-15R typically promotes basal-level proliferation [31,41]. Importantly, γc receptors co-regulate each other, which allows rapid synchronization through these cytokines, primarily via members of the signal transducer and activator of transcription (STAT) transcription factors [42]. 

Following a TCR signal, naïve T cells (T cell priming) and memory T cells (recall) rapidly downregulate IL-7Rα expression and upregulate CD25, the high-affinity receptor for IL-2 [42,43]. The expression of CD25 forms a highly stable IL-2R with much higher affinity for IL-2 than IL-15 [44,45]. Consequently, during activation (effector phase), memory T cells become unresponsive to IL-7 and IL-15 and heavily depend on IL-2 for survival and proliferation [31,46,47]. Thus, the γc family of cytokine receptors and associated cytokines regulate cell numbers of naïve and memory T cells via co-regulation as well as receptor affinity to allow QS-like rapid adaptation tailored to the need of the immune system at different stages and anatomical compartments.

#### 2.1.1. IL-2 Mediated QS-like Behavior of Effector and Regulatory T Cells

**Figure 1:**
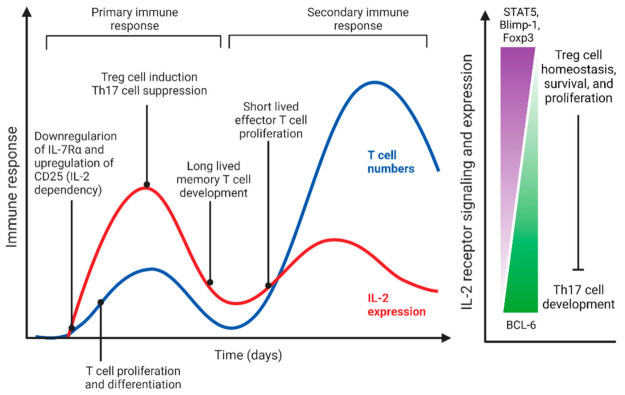
IL-2 as a density-dependent QS-like regulator of T cells. IL-2 and its receptor (CD25) are upregulated in T cells upon activation, leading to density-dependent changes and dose-dependent synchronization of the immune response. Low-dose IL-2 signaling leads to proliferation of early-activated T cells, their differentiation into effector cells, and development of memory T cells. High-dose IL-2 induces Treg cell activation and Th17 cell suppression through control of transcription factors and signaling.

IL-2 is a type I cytokine exerting trophic activity to control T cell proliferation and expansion [48]. IL-2 is expressed by T cells upon their activation and operates analogous to a quorum sensing autoinducer promoting autocrine/paracrine T cell expansion, survival, and apoptosis in a STAT3- and STAT5-dependent manner [40,49]. IL-2/IL-2R is less involved in the resting state homeostasis of memory T cells [39,40]. Interestingly, IL-2 is also critical for regulatory T cell (Treg) expansion and survival due to its exquisite dependence on CD25 signaling [50]. Treg cell activation tangentially regulates lymphocyte homeostasis via cytokines such as IL-2, IL-10, and transforming growth factor (TGF)-β by suppressing CD4^+^ and CD8^+^ T cell proliferative activity, irrespective of antigen specificities [51,52]. Thus, it is conceivable that the tissue levels of IL-2 are influenced by local cell density and, in turn, regulate local homeostatic expansion, for example of Tregs (Figure 1).

For instance, monoclonal antibody (mAb)-mediated neutralization of circulating IL-2 or IL-2R reduces the abundance and proliferation of Foxp3-expressing CD4^+^ CD25^+^ Tregs during the neonatal period, leading to loss of immune tolerance [53,54]. Additionally, experimental knockout of the downstream transcription factor of IL-2R (CD25), STAT5, results in a loss of tolerance in mice due to a Treg density decrease. Indeed, IL-2 signaling through the high-affinity IL-2R is pivotal in Treg homeostasis, survival, as well as immunosuppression through Foxp3 and CD25 expression [55,56]. The TCRαβ T cell lineage actively participates in IL-2 secretion, as Tregs remain unable to autonomously secrete the cytokine [57]. Therefore, Tregs ceaselessly ensnare exogenous IL-2 to promote their proliferation and selectively preclude the uncontrollable activation of other immune cells [58]. Notably, in mice, IL-2 deficiency and IL-2 receptor (IL-2R) α or β mutations result in lethal lymphoproliferative autoimmunity [59,60,61,62]. Evidence from humans also supports that dysregulation of Tregs can result in immune pathology, as shown by the immunodysregulation polyendocrinopathy enteropathy X-linked (IPEX) syndrome, where individuals show mutations of Foxp3 or, more rarely, CD25 [63,64]. 

Mounting evidence suggests that homeostatic regulation of effector and regulatory T cell populations can keep the development of autoimmune pathology in check under inflammatory conditions [65,66,67]. Recently, in vitro experimental studies and computer simulations supported the notion of density-dependent modulation of T cells via IL-2; thus, a QS-like mechanism mediated via IL-2 is positioned as a mediator of proliferation and population-level collective responses [68]. In silico modeling revealed that effector T cell density modulates the IL-2-mediated phosphorylation of STAT5 to further expand effector T cells during antigenic stimulation in a positive-feedback loop [69]. Interestingly, quantification of IL-2 kinetic rates established that transient fluctuations can result in pleiotropic pSTAT5-mediated effects enhancing Treg immunomodulation or promoting effector T cell survival. Notably, IL-2 scavenging by Tregs resulted in decreased STAT5 phosphorylation and the suppression of both weakly and strongly activated effector T cells [69]. Subtle IL-2 threshold fluctuations presumably promote complex IL-2R-mediated interactions abrogating T cell self-responsiveness or eliciting autoimmune pathogenesis.

The findings by these authors are consistent with a live-cell imaging study in microwell arrays examining the conditional differentiation of progenitor central memory T cells (pTCMs) during priming in OT-II TCR transgenic CD4^+^ T cells. Critically, the magnitude of naïve CD4^+^ T cell differentiation into memory precursors was optimal at a concentration greater than or equal to 30 cells per microwell [29]. Thus, density-dependent interactions collectively manifested due to a dynamic IL-2 and IL-6 regulatory interplay [29]. The authors speculated that novel mediator molecules potentially participate in the intricate differentiation cycle. 

#### 2.1.2. QS-like Behavior Mediated by IL-7 and IL-15 in Homeostatic Regulation of Naïve and Memory T Cells

IL-7 is an extracellular matrix-bound cytokine that is constitutively produced by cells in multiple tissues, including (i) stromal and epithelial cells in primary lymphatic organs (e.g., the bone marrow), where IL-7 is involved in lymphopoiesis; (ii) fibroblastic reticular cells in the T cell zone in secondary lymphoid organs; and (iii) cells in non-lymphoid organs including keratinocytes, mucosal epithelial cells, and even neurons [70,71,72,73]. CD8^+^ T cells surviving the contraction phase may convert into self-renewing long-lived memory cells through the action of IL-15 (reviewed in [36]). However, CD4^+^ T cells are less dependent on IL-15; therefore, additional factors may constrict the acquisition of long-lived capacities. Recent investigations have established that low clonal abundance favors naïve CD4^+^ T cell maintenance, activation, and survival of memory cell progeny [74]. Evidently, optimally diverse T cell repertoires must be homeostatically regulated to mount effective responses and facilitate long-lived memory CD4^+^ cell generation.

The IL-15R is composed of γc and a receptor chain shared between IL-15R and IL-2R known as IL-15R beta (IL-15Rβ). Both IL-2R and IL-15R are composed of an additional unique α chain that bestows the highest affinity for each of the cytokines: IL-2Rα (CD25) for IL-2 and IL-15Rα for IL-15 [75]. Importantly, IL-15Rα is unique in that it is expressed by the same cells that express IL-15 and functions to tightly bind IL-15 on the surface of this cell to present it in trans to T cells [75,76]. Thus, a T cell expressing the IL-15R-signaling component receives signals in trans provided by IL-15/IL-15Rα expressing neighboring cells [77,78]. Conventional memory T cells typically remain in a resting state where they chiefly respond to IL-7 and IL-15 signals to mediate their maintenance as well as long-term survival [31]. The homeostatic maintenance of the memory T cell pool is dictated by IL-7 availability and exhibits low spontaneous cell turnover [79,80,81,82]. 

Experimental evidence indicates that IL-7 maintains critical basal level proliferation of CD4^+^ Ag-specific memory T cells, especially under lymphopenic conditions [83]. Moreover, IL-7 alternatively induces long-term survival and reversion of effector Ag-specific CD4^+^ memory T cells to a resting state [84,85,86,87]. Altogether, local memory CD4^+^ T cells exhibit tight γc cytokine modulation, primarily via IL-7. In summary, the γc family of cytokines and receptors plays a critical role in the development, regulation, and homeostasis of T cells, operating as an autoinducer-like function endowing the adaptive immune system with a QS-like behavior by competing for γc availability and expression, mediated by members of STAT transcription factor family.

### 2.2. The Aryl Hydrocarbon Receptor (AhR) as a QS-like Regulator in Immune Cells

**Figure 2:**
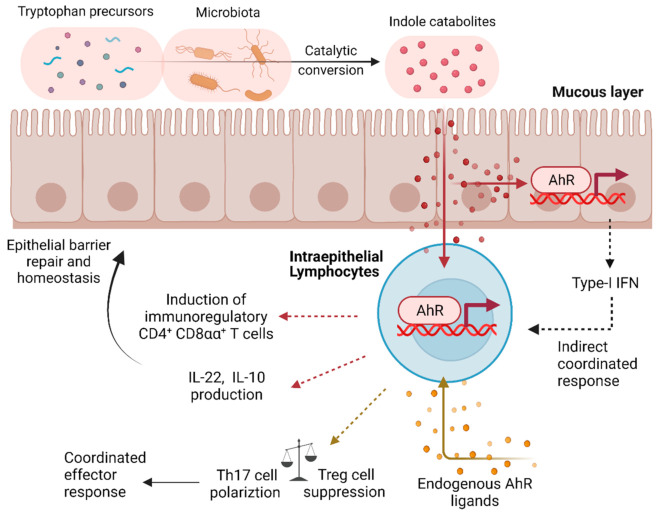
The aryl hydrocarbon receptor (AhR) is a QS-like regulator in immune cells. AhR expressed by immune cells (e.g., intraepithelial T cells) and non-immune cells (e.g., epithelial cells) senses exogenous and endogenous ligands, such as tryptophan metabolites produced by gut microbiota and orchestrates host transcriptional changes resulting in cytokine production and re-programming of T cells in response to environmental changes and altered metabolic demands.

The dynamic milieu of the immune microenvironment during healthy and disease conditions affords constant cellular adaptation. Homeostatic sensors integrate complex xenobiotic, metabolic, and endogenous stimuli into specific cellular responses. The transcription factor AhR is a highly conserved member of the basic helix–loop–helix/Per-ARNT-Sim (bHLH-PAS) homology sequence along with its competitive AhR repressor (AhRR) and its co-regulator AhR nuclear translocator (ARNT) [88,89]. Notably, AhR is mechanistically a transcription factor also exerting canonical ligand activation to sense a myriad of regulators in the environment such as diet, toxins, and microbiome or endogenous factors such as oxygen level and redox potential [90]. AhR is thereby polyfunctional, acting as a unique receptor and transcription factor activated directly through cognate ligand binding instead of upstream signal-transduction mediators [91]. Furthermore, AhR is expressed by many immune cells, including T cells, where it plays key roles in early T cell activation, differentiation, and effector functions [90,92]. Interestingly, a recent study indicated that the immune system can detect bacterial autoinducers via AhR, intercepting bacterial QS communications [93,94]. Taken together, as shown in Figure 2, AhR may allow immune cells to rapidly adapt to environmental conditions and can be viewed as a key sensor akin to a QS-like function, which may have evolved from allowing detection of microbial autoinducers by the human immune system to coordinate certain elements of its response at the population level. 

The mammalian digestive tract provides a unique environment to deliver QS signals to orchestrate host and microbiota crosstalk through several mechanisms that involve the immune system [95,96]. Along these lines, tryptophan metabolites produced by gut microbiota serve as AhR ligands in the digestive system [97,98,99], notably promoting host homeostasis by enhancing the intestinal epithelial barrier, motility, and hormone secretion via exerted anti-inflammatory effects [100]. A recent study uncovered that Lactobacillus reuteri activates the AhR to reprogram intraepithelial CD4^+^ T helper cells into CD4^+^ and CD8^+^ double-positive immunoregulatory T lymphocytes [99]. Indole-mediated interplay via AhR orchestrates host transcriptional alterations resulting in IL-22 and type I-IFN signaling to promote epithelial barrier repair during acute inflammatory episodes while additionally tuning intestinal homeostasis via IL-10 secretion [101,102,103,104,105]. These findings are consistent with a mouse model examining AhR agonist deprivation, as IL-22 impairment triggers inflammatory bowel disease [106]. Bacterial catabolite crosstalk thereby ostensibly confers homeostatically protective roles in and via the digestive system partially through AhR signaling.

The immune system regulates cell pool thresholds in response to physiological or unphysiological conditions via expansion or contraction of leukocyte populations, thereby maintaining a delicate steady-state equilibrium. Therefore, similar to the role of AhR in coordinating immune responses to environmental and bacterial cues, it is possible that the immune system has adopted this signaling pathway to also maintain a balance between immune pathology and immunoregulation, for instance, through coordinating the regulation between inflammatory Th17 cells and regulatory/non-inflammatory Treg cell population size [107,108]. Inherently, Treg/T_H_17 homeostatic dysregulations contribute to a spectrum of autoimmune, infectious, and cancerous conditions [109,110,111,112,113,114,115]. The underlying mechanism is not fully established; nevertheless, the AhR may play a pivotal role. IL-17/IL-22-secreting T_H_17 subsets express the AhR abundantly; contrarily, transcription is moderately low in Tregs and below detection in T_H_1, T_H_2, and naïve CD4^+^ T cells [116,117]. 

Compelling clinical studies examining ankylosing spondylitis found that the AhR agonist Semaphorin 4D induces T_H_17 polarization and inhibits Treg differentiation by downregulating Foxp3 expression [118]. Confirmatory transcriptome studies validated that AhR-dependent signaling regulates IL-17 and IL-22 secretion in human CD4^+^ T cells treated with T_H_17 inducing cytokines [119]. In a similar vein, in vivo studies examining AhR induction with a potent agonist, FICZ, revealed relapses in this experimental autoimmune encephalomyelitis (EAE) model due to exacerbated T_H_17 differentiation [120]. AhR activation via polycyclic aromatic hydrocarbons, prevalent in cigarette smoke, analogously exacerbates arthritis via increased T_H_17 polarization in mice [121]. The governing molecular pathway(s) remain incompletely understood. However, AhR dampens STAT1 and STAT5 signaling pathways, dichotomous regulators of T_H_17 transcriptional programs [122]. Notably, AhR and STAT3 upregulate Aiolos in T_H_17 polarizing microenvironments to preclude apoptosis via Bcl-2 activity [123,124]. The authors discovered that Aiolos silences the Il2 locus, thus facilitating T_H_17 differentiation in vitro and in vivo [124]. Altogether, we posit that AhR-mediated activity may induce homeostatic disturbances promoting autoimmune pathogenesis via Treg/T_H_17 cell pool imbalances in the absence of commensal organisms.

### 2.3. Bystander Activation of T Cells: QS-like Acute Phase Response of Specific T Cell Subsets

Bystander activation is a mechanism where T cells specific for unrelated antigens proliferate and/or are activated in various disease conditions independent of TCR signaling [125,126,127]. Bystander proliferation was first reported by Tough et al. in 1996 as a massive expansion of CD8^+^ T cells in the absence of detectable TCR signaling after heterologous virus infection [128]. Subsequent studies, however, showed that the extent of bystander activation may have been overestimated initially and that the majority of T cells activated by infections are virus-specific [129,130]. Nevertheless, bystander activation is thought to be a mechanism that may be beneficial or detrimental to the host depending on the circumstances [127]. 

Bystander activation is primarily observed by CD8^+^ memory T cells (T_mem_) and occurs rapidly during the earliest phase of infection [127]. It is driven by proinflammatory cytokines, including type I interferons (IFNs), IL-12, IL-15, and IL-18, and appears to be mediated independently of cognate TCR signaling [127]. Bystander activation by CD4^+^ T cells is less well understood but is also centered on T_mem_ subsets and seems to be less efficient due to decreased expression of CD122 [126]. As pointed out earlier, bystander activation is primarily observed under infectious conditions, and it was first described during virus infections. However, bystander activation was also demonstrated during bacterial infections and by bacterial components such as LPS [125,126]. Indeed, Toll-like receptors (TLRs) or other innate-like receptors such as NKG2D have been reported to mediate bystander activation [125,126]. Bystander activation can result in T cell proliferation, cytokine expression, and direct cytolysis of infected cells [127] and may therefore be beneficial during infection. However, bystander activation may be detrimental in certain conditions such as chronic infections or autoimmune diseases [126]. 

Taken together, bystander activation shows some similarities with QS and encompasses certain aspects of it, but it seems to be centered on specific T cell subsets (e.g., T_mem_) and to occur only during particular circumstances, such as infections and chronic disease conditions. Moreover, QS-like behavior of adaptive and innate immune cells seems to conceptually separate from bystander activation by focusing on synchronizing the behavior of communal cell populations, whereas bystander activation seems to be focused on eliciting a (non-specific) effector response to support the earliest phase of anti-microbial immunity.

## 3. QS-like Regulation of Myeloid Cells and the Innate Immune System

### 3.1. QS as a Myeloid Lineage Modulator: Population Density of Tissue-Resident Macrophages Contributes to Spatiotemporal Regulation

Tissue-resident macrophages are phagocytes responsible for maintaining tissue homeostasis and repair, defending against pathogen invasion, and removing extracellular debris such as apoptotic cells and toxic metabolic products [131,132]. Current evidence supports that most tissue-resident macrophages may arise from embryonic precursors seeding during development as opposed to primarily deriving from hematopoietic stem cells [133,134,135,136]. Consequently, the classic paradigm postulating macrophage replenishment via circulating monocytes has shifted to incorporate embryonic precursor self-renewal based on dynamic tissue regulation. 

Macrophages exhibit prolific physiological plasticity in response to endogenous and exogenous stimuli [137]. Consequently, phenotypic variation results from diverging cellular differentiation patterns partly constrained by microenvironmental parameters. Historically, macrophage polarization nomenclature comprises the classically (M1) and alternatively activated (M2) populations distinguished by unique activation markers [138]. M1 polarization is functionally associated with inflammatory (post-infectious pathogenesis) and microbicidal activity, whereas the M2 phenotype confers immunomodulatory properties mediating inflammation resolution [139]. Intricate phenotypic distributions coexist in specific tissues; therefore, regulatory mechanisms spatiotemporally exert homeostatic balance.

Compelling evidence suggests that regulatory mechanisms akin to QS serve to reestablish the steady-state concentration of macrophages following infectious episodes and injury [15]. These findings are also consistent with seminal research investigating the effects of diphtheria toxin (DT)-mediated selective depletion of liver-resident macrophages known as Kupffer cells (KCs) in a humanized mouse model [140]. The authors identified that congregating macrophages or myeloid cells acquire transcriptionally homologous tissue-resident macrophage signatures, including self-renewal capacities, within 15 days of colonization [140]. Additionally, acute acetaminophen (N-acetyl-p-aminophenol) overdose stimulated the self-renewal of tissue-resident macrophages following a marked reduction in the KC population [141]. In this case, the recruitment of circulating monocytes resulted in a transcriptionally distinct phenotype that failed to contribute to KC replenishment. The exact mechanism(s) governing the spatiotemporal regulation of tissue-resident macrophage density following threshold diminishment and the transcriptional acquisition of self-renewing phenotypes by monocyte-derived macrophages remain unsolved. However, these data collectively suggest that the steady-state concentration of macrophages is dynamically regulated in response to infection and collateral tissue damage. Consequently, it is conceivable that QS-like mechanisms could regulate tissue homeostasis of macrophage populations.

### 3.2. Apoptotic Metabolite Release as a Putative QS-like Mechanism Governing Macrophage Density

**Figure 3:**
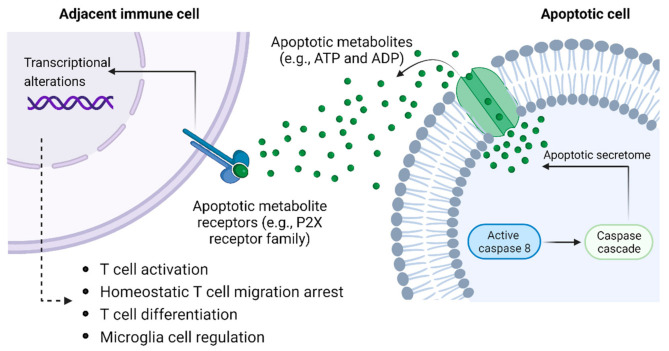
Apoptotic metabolites as QS-like regulators of immune cell function. Apoptotic metabolites released by cells undergoing apoptosis can induce transcriptional programs similar to QS to synchronize and coordinate T cell responses (and potentially those of other immune cells) in the proximity to regulate dynamic tissue responses to infection and tissue damage. Important known apoptotic metabolites include ATP and ADP, which signal through the P2X receptor family involved in inducing T cell activation, differentiation, and migratory changes.

Groundbreaking mechanistic studies indicate that apoptotic metabolites orchestrate transcriptional alterations facilitating inflammation resolution, cell proliferation, and tissue regeneration in healthy neighboring cells to restore a homeostatic state [142]. Secretome profiling analyses identified six conserved metabolites endowing a fundamental signaling role: AMP, GMP, creatine, spermidine, glycerol 3-phosphate, and ATP [142]. The authors detected diverse apoptotic metabolites in macrophage and lymphocyte cell pellets, although caspase-mediated activation of pannexin-1 hemichannels selectively regulated molecular release under intact membrane conditions. These data collectively challenge the paradigm construing programmed cell death pathways as metabolically inert. Contrarily, the apoptotic secretome is selectively regulated to coordinate collective gene expression in local microenvironments. We postulate that signaling via apoptotic metabolites induces threshold-specific transcription programs similar to QS. Elevated apoptotic metabolite concentrations can conceivably modulate synergistic macrophage responses in dynamic tissue parameters such as collateral tissue damage and infectious disease [143]. Moreover, metabolites that are secreted or diffuse from apoptotic cells, such as ATP and ADP, can regulate both the adaptive and innate immune system simultaneously to coordinate the immune response and immune cell homeostasis [144,145,146,147,148], thus endowing it with a QS-like mechanism to regulate tissue populations in response to local cell death (Figure 3). 

In the central nervous system (CNS), microglia mechanistically operate as resident macrophages to safeguard and support neuronal functions [149]. Nevertheless, macrophages and microglial cells differ in calcium fluctuation responses, expressed biomarkers, and inflammatory profiles following traumatic events such as brain ischemia [150,151,152,153]. In the face of inflammatory or pathological insults, CNS microglia and macrophages establish the first line of defense via innate and adaptive immune components [154]. Uncontrolled microglial activation and homeostatic dysregulation contribute to CNS disorders. Indeed, unresolved chronic inflammation can result in neuronal and glial damage. 

Recently, a multicolor fluorescence mapping reporter system using confocal microscopy established that expansion of microglia populations increased proximal to the site of facial nerve transection in mice; however, the pre-injury network density of microglial cells was homeostatically restored [155]. The authors also found that microglial self-renewal rates corresponded to cortex, cerebellum, and hippocampus morphological proliferation in healthy mice. We posit that the spatiotemporal regulation of microglial cells in response to injury is a QS-like mechanism, potentially guided via apoptotic metabolite release. Nonetheless, the mechanisms remain in question as the collective interaction of microglia and macrophages to restore and maintain a proper tissue density is unknown. 

### 3.3. QS-like Density-Dependent Polarization of Macrophages

Recent evidence indicates that a density-dependent QS-like mechanism controls macrophage polarization and the magnitude of the inflammatory response [156,157,158]. For instance, it was reported that follicular micro-injuries caused by hair plucking trigger a concerted macrophage-mediated regeneration of resting cells in mice [157]. Subsequent molecular and genetic analyses unraveled a two-step mechanism mediated by the follicular secretion of CC-chemokine ligand 2 (CCL2) to induce the dermal recruitment of TNF-expressing macrophages to promote local regeneration [157]. Interestingly, macrophages collectively assess the magnitude of hair follicle injuries to elicit all-or-none responses [157]. Macrophage accumulation and dissemination could therefore serve as a mechanism orchestrating local keratinocyte apoptosis to sustain a coordinated regenerative anagen phase. Along these lines, a single-cell tracking study determined that the lipopolysaccharide-induced bimodal phenotypic partitioning of primary macrophages is contingent on cell density and concerted gene expression [25]. These observations were TNF-independent, as distinct polarizations originated due to resting-state density priming, a pre-programmed response coined ‘quorum licensing’ by the authors. Hence, cell density information potentially regulates the collective activation of macrophages.

Colony-stimulating factor 1 (CSF-1) has been postulated as a central regulator of macrophage density at a steady state [6,159]. For example, stromal cells and endoneurial fibroblasts secrete CSF-1 to modulate macrophage survival, proliferation, as well as differentiation in vivo [160,161]. However, the role of CSF-1 remains controversial as the interpretation of results can be confounded by the pleiotropic effects exhibited by pharmacological or antibody-mediated CSF-1 receptor (CSF-1R) blocking. The ligands CSF-1 and interleukin-34 (IL-34) share the promiscuous CSF-1R; therefore, receptor neutralization may result in local and systemic off-target effects that may have confounded the interpretation of the results. Similarly, CSF-1R neutralization in vivo leads to physiological increases in CSF-1 levels, thus presumably interfering with the monoclonal antibody’s activity [162]. Additionally, alternative splicing and differential proteolysis render three CSF-1 homodimeric isoforms which may differ in affinity and function: a cell-surface glycoprotein (csCSF-1), a secreted glycoprotein (sgCSf-1), and a proteoglycan (spCSF-1) [163]. Notably, recent evidence revealed that spCSF-1 induces macrophage activation and neuronal damage, whereas csCSF-1 attenuates macrophage-mediated neuropathy in a mouse model for Charcot–Marie–Tooth type 1X disease [164]. Additional studies are required to further dissect the intrinsic role of each CSF-1 isoform as a QS-like cell density regulator in macrophages.

### 3.4. Intersection of Microbial QS with Regulation of Myeloid Cells and Tissue Macrophages

Macrophages undergo multifaceted regulation during the course of infectious diseases. In the lung microenvironment, alveolar macrophages are sentinel cells responsible for maintaining lung homeostasis, clearing cellular debris, and protecting against pathogen invasion [165,166]. However, pathogens have evolved exquisite mechanisms to evade immune surveillance, survive in hostile microenvironments, and subvert host responses. Facultative intracellular bacteria such as the pulmonary pathogen *Mycobacterium tuberculosis* (*Mtb*) can establish a permissive milieu conducive to disease persistence and systemic dissemination [167].

Apoptosis is an innate host-protective mechanism precluding *Mtb* propagation (reviewed in [168,169]); in turn, *Mtb* can differentially manipulate the timing and mode of cell death in infected alveolar macrophages [170,171,172]. Murine and human macrophage models showed that virulent *Mtb* strains dampen apoptosis while favoring necrotic niches associated with granuloma formation, bacterial replication, as well as uncontrolled dissemination [173,174]. Compelling evidence also indicates that human macrophage coordination at high cell density (2 × 10^5^ cells/well) profoundly suppressed *Mycobacterium bovis* pathogenesis and growth of a live-attenuated strain known as BCG [156]. The effects were independent of mycobacterial uptake, multiplicity of infection, extracellular medium acidification, nitric oxide production, or paracrine stimulation via cytokines, as the study considered such confounding variables. We propose that QS-like collective macrophage regulation may be mediated by apoptotic metabolites and promotes cellular synergism. Likewise, TLR activity emerges as an alternative mechanism regulating QS-like responses in macrophages and dendritic cells (DCs). Viral sensors such as TLR3 provoke phenotypic alterations in DC biology, including type I IFN secretion for microenvironmental propagation [175]. A compelling study suggests that optimal DC activation is mediated by a quorum of type I IFN-secreting cells at the lymph node level [176]. The authors also found that collective DC activation is essential to mount robust innate and adaptive immune responses in the lymph node. Of note, activation of CD4^+^ T cells requires a minimum threshold of approximately 85 antigen-presenting DCs in lymph nodes [177]. Similarly, TNF secreting populations induce concerted macrophage activation and coordinate responses at the population level in response to LPS stimulation of TLR4 [178]. Thus, TLR-mediated signaling may induce QS-like regulation to mount synergistic responses geared towards eliminating foreign invaders.

## 4. Clinical Implications

### 4.1. Exploring QS-like Regulation of T Cells for Inflammatory Diseases and GvHD

Coordinating T cell responses via γc receptors and cytokines can act as a QS-like mechanism for the regulation of T cells under inflammatory conditions. For example, IL-2 can promote cellular responses akin to QS. Of note, IL-2 has been investigated as a viable therapeutic modality to treat underlying autoimmune pathologies, cancer, hypersensitivity responses, and allograft rejection [50,179,180,181]. 

Currently, the implications of IL-2 as a QS-like mediator have not been fully explored, yet this area may hold promise for treatment purposes. Along these lines, pharmacokinetic studies investigating mice and human responses to low-dose IL-2 (LD IL-2) have identified marked increases in Treg activation and suppressive activities [182,183,184,185]. In NOD mice, treatment for five days with LD IL-2 prevented the onset of type 1 diabetes by increasing pancreatic Treg populations [186]. Randomized placebo-controlled phase I and II clinical trials exposed six patients to daily placebos or IL-2 doses of 0.33, 1, or 3 million international units (MIU) for a 5-day course (reviewed elsewhere [50]). The results showed that IL-2 therapy facilitated a dose-dependent increase in CD4^+^ and CD8^+^ Treg cells in the absence of detrimental adverse reactions [187]. Similarly, modified IL-2 with extended in vivo half-life (termed IL-2 mutein) was shown to selectively activate and expand regulatory T cells [179]. 

Furthermore, clinical trials investigating the effect of LD IL-2 in active systemic lupus erythematosus (SLE) have been successful in mitigating refractory phenotypes to standard therapy [188]. Severe SLE manifestations can be attenuated with an initial IL-2 dosage of 1 MIU augmented to 3 MIU for five days; consequently, Treg populations increased while anti-dsDNA antibody concentrations were reduced [189]. The clinical feasibility of LD IL-2 has been extrapolated as a novel treatment for autoimmune alopecia areata [190]. The results of the study showed that low-dose recombinant IL-2 induced a marked increase in CD4^+^ CD25^+^ FoxP3^+^ Treg recruitment, accompanied by a decrease in effector CD8^+^ T (T_eff_) cells from scalp biopsies [190]. Treatment resulted in severity of alopecia tool scores decreasing from 82 to a baseline of 69, therefore manifesting as partial hair regrowth in 4 out of 5 patients at six months with no reported adverse reactions [190]. These results collectively suggest a QS-like mechanism for regulating Tregs and effector T cells in autoimmune diseases. 

LD IL-2 also conferred beneficial effects for the treatment of chronic graft versus host disease (GVHD) after allogeneic hematopoietic stem cell transplantation [191,192]. LD IL-2 treatment induced the homeostatic regulation of CD4^+^ T cells following transplantation as well as an increased expansion of Tregs [193,194]. In allogeneic hematopoietic stem cell transplantation patients, chronic myelogenous leukemia was significantly ameliorated upon LD IL-2 administration combined with Treg infusion [195]. LD IL-2 can alternatively elicit Treg expansion and confer a protective role in clinical manifestations of food allergy in mouse models [196]. Thus, regulation of cell density via IL-2, for example, for Tregs, may be an example of exploiting the mechanism of QS for therapeutic benefits. A critical question that has not been addressed currently is whether increased cell density translates to changes in function in these systems.

We posit that dysregulation of one T cell subset (i.e., Treg cells) results in disturbances of other T cell subsets, for example, by promoting effector T cell activities in inflammatory/autoimmune diseases. Density dysregulation may therefore be a key feature in a spectrum of adverse immunological conditions. The exact pathophysiological mechanism(s) remain incompletely resolved; however, it appears that mediators, such as IL-2, can regulate collective responses in a QS-like mechanism and that their disruption may foster tissue pathology. Accordingly, threshold parameters in IL-2, for example, will dictate their immunomodulatory potential as a novel strategy to treat pathological inflammatory conditions. In addition, other QS-like mediators, such as AhR agonists, may have similar or even more potent effects on T cell regulation. Thus, we propose that exploration of QS-like modulation in immune cell populations may hold as of yet untapped potential. 

### 4.2. QS-like Modulation of T Cells for Cancer Immunotherapy

Oncogene expression and tumor suppressor deactivation are among the mechanisms mediating carcinogenesis, an uncontrolled cellular proliferation eluding cell-regulatory mechanisms [197]. Surgical excision of tumors is typically the primary modality for solid cancers, yet surgical or other interventions such as radiation or chemotherapy are often futile in advanced cancer stages, where most malignant tumors have broken through organ confines, invaded other tissues, and metastasized [198]. Immunotherapies have emerged as promising adjunct or salvage alternatives for cancers that are difficult to treat with conventional medical therapies, such as metastatic malignant melanoma. Ultimately, immunotherapies could selectively target cancer cells while mitigating residual side effects associated with traditional treatment regimens. For example, checkpoint inhibition, adoptive cell therapy, and bivalent antibodies are clinically successful immunotherapies in combating otherwise incurable malignancies.

In contrast to harnessing inflammatory conditions with LD IL-2, high-dose IL-2 (HD IL-2) formulations were used to combat malignant tumors, for example in melanoma and renal cancer patients [199,200,201]. In malignant melanoma patients, drug administration resulted in a durable overall remission in 16% of the cases with reported toxicities such as hypotension and secondary to underlying capillary leak [202]. Moreover, patients exhibited a tumor regression rate of 20% in renal cell carcinoma trials [203]. Collectively, these data indicated that HD IL-2 promoted a polyclonal expansion of effector T cell subsets to ameliorate anergy and mediate tumor regression. Of note, experimental evidence indicates that exogenous HD IL-2 abrogated CD25^+^ FoxP3^+^ Treg-induced immunosuppression [204]. Commensurate increases in effector T cell population density circumvent Treg attenuation; thus, the QS-like effects of IL-2 may emerge as a viable concept in immuno-oncology. 

### 4.3. Implications of QS for Treatment of Infectious or Inflammatory Diseases via Regulation of Macrophage and Myeloid Cells

Therapeutically, the feasibility of exploiting QS-like signaling for host-directed therapies to optimize antimicrobial immunity, ameliorate immunopathology, and collateral tissue damage has yet to be determined. However, QS-like regulation of density-dependent macrophage responses may hold untapped potential for improving difficult-to-treat infectious diseases, such as multidrug-resistant tuberculosis. Macrophages may be instructed to collectively polarize, thus resulting in enhanced pathogen neutralization. Further research with diverse pathogens may reveal novel mechanisms in macrophage biology, as, for example, apoptosis metabolite thresholds are unknown for specific microenvironments. Furthermore, compelling preliminary data suggest that local administration of select metabolite cocktails dampens inflammatory arthritis and lung graft rejection in mouse models [142]. Taken together, research to harness macrophage QS may have the potential to lead to a new generation of host-directed therapies.

## 5. Concluding Remarks

Spatiotemporal modulation of adaptive and innate immune cell density following infectious episodes, acute injury, and immunopathology has profound consequences for immune responsiveness and homeostasis. QS-like signaling has the potential to guide collective tissue immune responses, for example, via density-dependent synergistic interactions. Furthermore, the mammalian digestive tract poses a unique QS-sensing portal orchestrating host–commensal crosstalk.

To further elucidate the biological complexities of QS, complementary approaches will benefit from integrating biological models with bioinformatics and omics studies in combination with computational models. The application of these approaches to QS in bacteria has revealed novel insights, for example to decipher biofilm formation [205,206]. Similarly, theoretical considerations and mathematical modeling of QS for effector and regulatory T cells have provided novel insights and broadened our conceptual understanding of immune QS and QS-based decision making [207,208,209]. It is hoped that these approaches accelerate our comprehension of QS in the immune system and facilitate discovery of regulatory mechanisms and autoinducers. 

We posit that QS-like mechanisms endow a regulatory role in immune homeostasis and communal effector responses. Therefore, we anticipate that cell pool dysregulations may etiologically contribute to immune aberrations. Compelling evidence revealed that AhR-mediated signaling induces robust polarization of the inflammatory T_H_17 subset while selectively downregulating Foxp3 expression to preclude Treg differentiation. Subsequently, Treg/T_H_17 cell pool imbalances might conceivably foster autoimmune pathogenesis. Future studies will demonstrate the clinical feasibility of host-directed therapies tailored to QS-like mechanisms in human disease conditions.
